# Geometry-Based Stochastic Channel Model for Vehicle-to-Vulnerable Road Users Communications in Critical Scenarios

**DOI:** 10.3390/s26196281

**Published:** 2026-10-03

**Authors:** Ibrahim Rashdan, Fabian de Ponte Müller, Stephan Sand, Giuseppe Caire

**Affiliations:** 1 German Aerospace Center (DLR), Institute of Communications and Navigation, 82234 Oberpfaffenhofen, Germany; fabian.pontemueller@dlr.de (F.d.P.M.); stephan.sand@dlr.de (S.S.); 2 Department of Electrical Engineering and Computer Science, Technical University of Berlin, 10623 Berlin, Germany; caire@tu-berlin.de

**Keywords:** GSCM, WINNER, radio channel model, VRU, V2P, V2VRU, parameterization, validation

## Abstract

Direct vehicle-to-vulnerable road user (V2VRU) communication can prevent accidents by enabling mutual awareness between vehicles and vulnerable road users (VRUs) such as pedestrians and cyclists. A prerequisite for developing reliable V2VRU communication systems is having a channel model that captures the main propagation characteristics of this specific link type. In this work, a measurement-based parameterization and validation of a WINNER-type V2VRU geometry-based stochastic channel model (GSCM) is presented at 5.2 GHz, based on single-input single-output (SISO) wideband channel measurements in urban environments. The three most critical accident-prone scenarios involving pedestrians and cyclists are considered. The large-scale parameters (LSPs), path loss, shadow fading, delay spread, K-factor, and angular spreads, are estimated from the measurement data and fitted to log-normal distributions. The results reveal propagation characteristics that differ substantially from those reported for vehicle-to-vehicle (V2V) channels, including significantly higher path loss exponents under obstructed conditions and distinct LSP correlation structures. The proposed model is validated statistically and further assessed through link-level simulations. The simulations show that commonly used V2V channel models fail to reproduce the error floor observed in the measured V2VRU channels, which highlights the importance of using dedicated channel models when evaluating V2VRU safety applications.

## 1. Introduction

In recent decades, road traffic has increased enormously due to the rapid growth of population and cities. One major drawback of this expansion is the increase in road traffic accidents. According to the World Health Organization, traffic accidents take about 1.35 million lives and cause more than 50 million injuries globally each year with an associated governmental cost of about 3% of the gross domestic product (GDP) [1]. Vulnerable road users (VRUs), i.e., pedestrians, cyclists and motorcyclists, account for almost half of road victims. Based on accident statistics reported in [2,3], the most critical pre-crash scenarios that involve pedestrians and cyclists occur in urban environments. In these scenarios, the driver’s visibility of pedestrians and cyclists can be blocked by buildings at intersections or by parked vehicles along the roadside. Currently, driver assistance systems and automated vehicles only rely on their own perception sensors to detect and locate other surrounding traffic participants. However, radar sensors, laser-scanners, and camera-based systems have one critical limitation. They require direct sight of other road users. Additionally, light-based systems show poor performance under adverse weather or lighting conditions [4,5,6]. One way to overcome this limitation and obtain 360° awareness is to use a communication technology to directly exchange information between vehicles and VRUs [7,8]. Using vehicle-to-VRU (V2VRU) communication, also called vehicle-to-pedestrian (V2P) communication, can improve mutual detection, localization, and tracking of both vehicles and VRUs and generate appropriate warnings to prevent accidents.

V2VRU communication is part of vehicle-to-everything (V2X) communication, which also includes vehicle-to-vehicle (V2V) and vehicle-to-infrastructure (V2I) communications. V2X communication has the potential to increase the safety and efficiency of transportation systems. There are two main technologies that enable V2X communication, i.e., WiFi-based and cellular-based technologies. Dedicated short-range communication (DSRC) in the US or ITS-G5 in Europe are IEEE 802.11p-based technologies, and were developed over two decades ago. The 3GPP consortium is incorporating V2X communication capabilities into their long term evolution (LTE) standard under the name LTE-V2X or cellular-V2X (C-V2X) communication. Moreover, IEEE 802.11bd [9] and cellular 5G new radio (NR) V2X [10] technologies are expected to support V2X applications that require lower latency, higher reliability, and higher data rate.

VRU protection is a safety application that demands high reliability from all its subcomponents. Therefore, the V2VRU communication link must also be extremely reliable. A reliable communication link efficiently transmits data packets within the relevant range while maintaining a very low probability of packet loss. Designing and testing highly reliable communication systems requires a comprehensive understanding of propagation conditions, represented by appropriate channel models.

Channel models can be broadly divided in three categories: deterministic, stochastic, and geometry-based stochastic channel models (GSCMs). The two main factors that influence the decision of which modeling approach to follow are the complexity and accuracy of the model. Taking the non-stationarity nature of the V2VRU channel into account, a deterministic approach can provide a highly accurate and realistic channel model. However, it is site-specific and requires intensive and time-consuming computations. Stochastic models, on the other hand, have relatively low complexity, which makes them easy to use. Yet, they are inadequate to produce realistic models for non-stationary channels [11]. Combining the geometrical and statistical elements of the deterministic and stochastic models, GSCMs provide accurate geometrical relations with a reduced computational cost. Moreover, GSCMs are found to be well suited for non-stationary environments [12]. Therefore, we choose to follow the GSCM approach in modeling the V2VRU channel in this work. The two main types of GSCMs are the European cooperation in science and technology (COST 2100) [13] and the wireless world initiative for new radio (WINNER II) [14]. Since the COST 2100 channel model, on the one hand, is cluster-centric, it requires drawing the distribution of the scattering clusters from the measurement data. Unfortunately, our collected data are based on single-input single-output (SISO) measurements, which limits the ability to accurately acquire such distributions. On the other hand, the WINNER-type channel model is user-centric, i.e., the placement of the scatterers in the simulated propagation environment is based on the large-scale parameters (LSPs). These LSPs can be estimated based on the measurement data. Therefore, the WINNER-type channel model is chosen in this work.

Several channel models were proposed for V2V and V2I communications [15,16,17,18,19], and this area has continued to receive attention in recent years [20,21,22,23]. However, compared with V2V and V2I communications, V2VRU channel characterization remains limited. Existing V2P studies have investigated individual propagation characteristics such as path loss, fading, correlation, or first-order channel statistics [24,25,26,27,28].

There are only a handful of recent studies focused on modeling different aspects of the V2VRU channel. The authors in [24] performed V2P channel measurements at 3.8 GHz with a bandwidth of 200 MHz. Based on the measurements in the line of sight (LoS) scenario, the authors presented a two-ray path loss model for the strongest path contribution, and log-distance path loss model for the path loss of discrete scatterers. Normal and Ricean distributions for large-scale fading (LSF) and small-scale fading (SSF) were also reported. However, the proposed GSCM is limited to LoS conditions and does not report delay spread, angular spreads, or spatial correlations of the LSPs. The authors extended this work to a LoS/Non-LoS (NLoS) scenario in [25], where a multi-slope log-distance path loss model is proposed.

The authors in [26] investigated the autocorrelation function (ACF) of the V2P channel in LoS and partial LoS scenarios. According to their findings, the channel decorrelates rapidly in time domain. In [27], the authors investigated the first-order characteristics of the V2P channel at 5.8 GHz in a business district environment. The K-factor (KF) was calculated for different locations of the transmitter (Tx) and receiver (Rx) antennas and vehicle speeds. However, their work was based on narrowband received signal strength indication (RSSI) measurements which provide only limited information about the propagation conditions. The path loss was studied in [28] based on narrowband channel measurements in LoS when the pedestrian is stationary as well as when the pedestrian is moving parallel to the road. However, the measurements are based on received signal power rather than wideband channel sounding, which precludes the estimation of delay spread and angular spreads that are needed for a GSCM parameterization.

Table 1 summarizes the existing V2VRU measurement studies and positions this work relative to them.

To the best of our knowledge, a measurement-based WINNER-type GSCM parameterization covering the LSPs and their spatial and cross-correlations for critical urban V2VRU scenarios with LoS, Obstructed LoS (OLoS), and NLoS transitions has not been reported.

The main contributions of this work are as follows:

1. A SISO wideband V2VRU channel measurement campaign is conducted at 5.2 GHz in an urban environment, covering the most critical accident-prone scenarios involving pedestrians and cyclists. The considered scenarios include conditions where the line-of-sight between the vehicle and the VRU is obstructed by buildings and parked vehicles.
2. V2VRU-specific propagation characteristics are identified and quantified from the measurement data, including path loss behavior under low VRU antenna height conditions, LSP distributions, and their spatial correlations. The estimated parameters reflect the distinct propagation effects introduced by the low VRU antenna height and frequent LoS obstruction by parked vehicles—conditions that are not represented in existing V2V and V2I channel models and for which, to the best of our knowledge, no comparable measurement-based WINNER-type parameterization has been reported for the critical urban V2VRU scenarios considered here.
3. A measurement-based parameterization of a WINNER-type GSCM is provided for the considered V2VRU scenarios. The estimated model parameters are used as input to the QuaDRiGa channel simulator and validated by comparing the statistics of the simulated channels with those of the measured channels.
4. Through link-level simulations, it is shown that standard V2V tapped-delay line channel models fail to capture the error floor observed in the measured V2VRU channels. This underscores the importance of using a dedicated and realistic channel model when evaluating V2VRU safety applications.

The scope of the proposed parameterization is limited to the considered urban scenarios, frequency range, and measurement configuration.

It is worth clarifying why existing V2V and V2I channel models are not directly applicable to V2VRU scenarios. The most immediate difference is the antenna height: in V2VRU links, the VRU antenna is typically mounted below 1.5 m, which leads to stronger ground reflections and more pronounced diffraction losses around parked vehicles compared to vehicle-height antenna configurations. The practical impact of this can be seen directly in the measurement results of this work, where the estimated path loss exponent for the obstructed segment of Scenario 1 reaches 6.9, which is well above the values of 3–4 typically reported for urban V2V NLoS scenarios [15,16,17]. Beyond path loss, the K-factor statistics and angular spreads estimated here also differ markedly from V2V counterparts. The 3GPP channel models for V2X communication (TR 37.885, TR 38.901), while useful as a system-level baseline, assume vehicle-height antennas on both sides of the link and do not account for these effects. Using the parameters of these models for a V2VRU link would therefore produce channel realizations that do not reproduce the measured propagation conditions, leading to an unreliable assessment of system performance.

The remainder of this paper is organized as follows. Section 2 describes the measurement campaign. Section 3 briefly describes the estimation and tracking of the multipath components (MPCs) parameters. In Section 4 the channel model parametrization, implementation and validation methods are discussed. Section 5 presents the results followed by the conclusion.

## 2. Measurement Campaign

**Figure 1:**
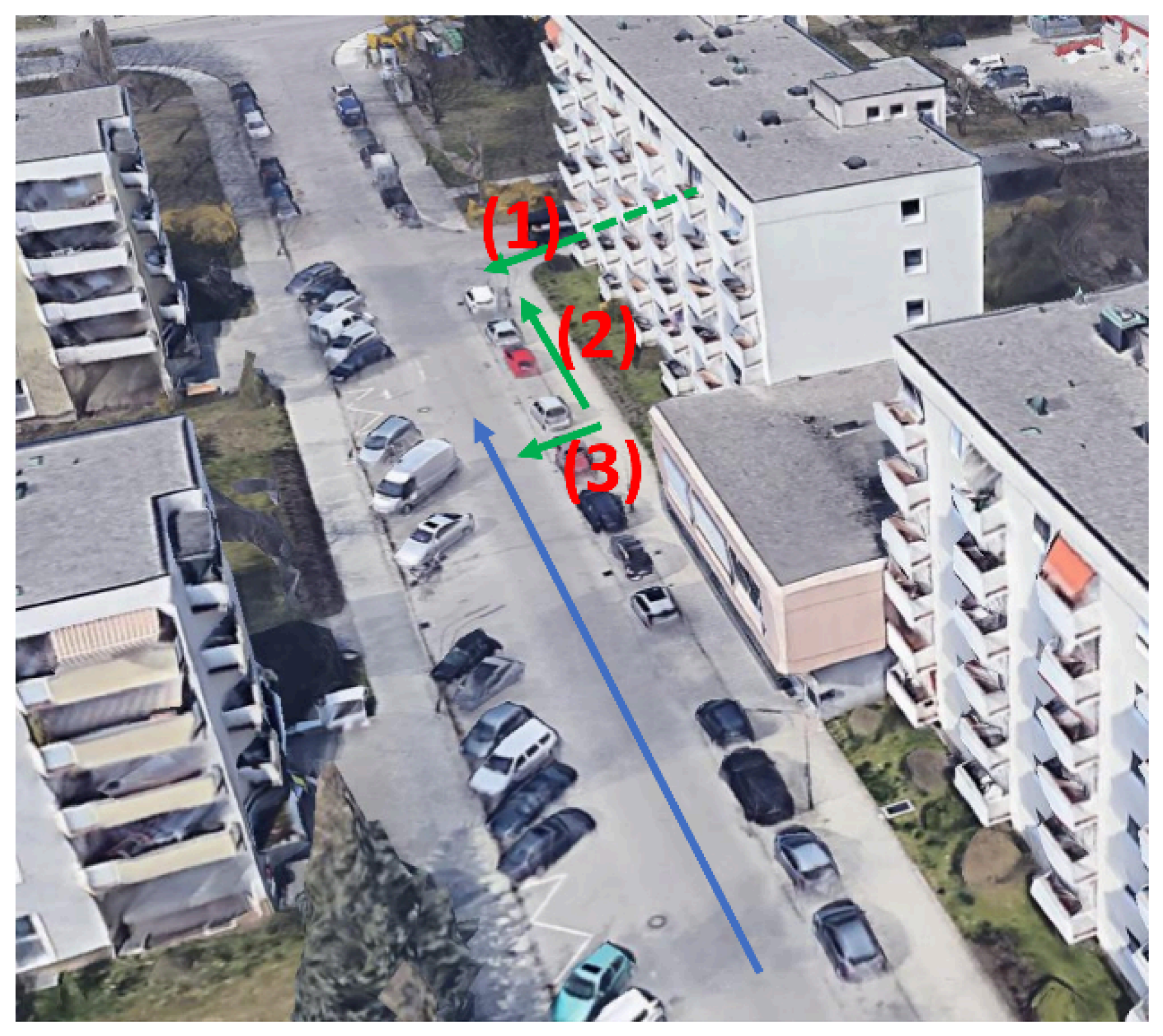
Aerial view of the measurement scenarios in the urban environment, showing the trajectory of the vehicle (in blue) and the trajectory of the cyclist/pedestrian (in green, labeled (1)–(3) by scenario number) towards the imaginary collision point. (Google Earth 2018 Geobasis-DE/BKG).

Channel measurements were conducted in October 2018 in the city of Germering near Munich. In this work, the channel measurements in Göthestraße will be analyzed (see Figure 1), where three–six story buildings are lined up along the street on one side, while being separated by a green area on the other side. The street consists of one lane for each direction with parked cars on both sides. It is 12 m wide, with 3 m wide sidewalks. More details on the measurement campaign can be found in [29].

### 2.1. Measurement Systems

**Figure 2:**
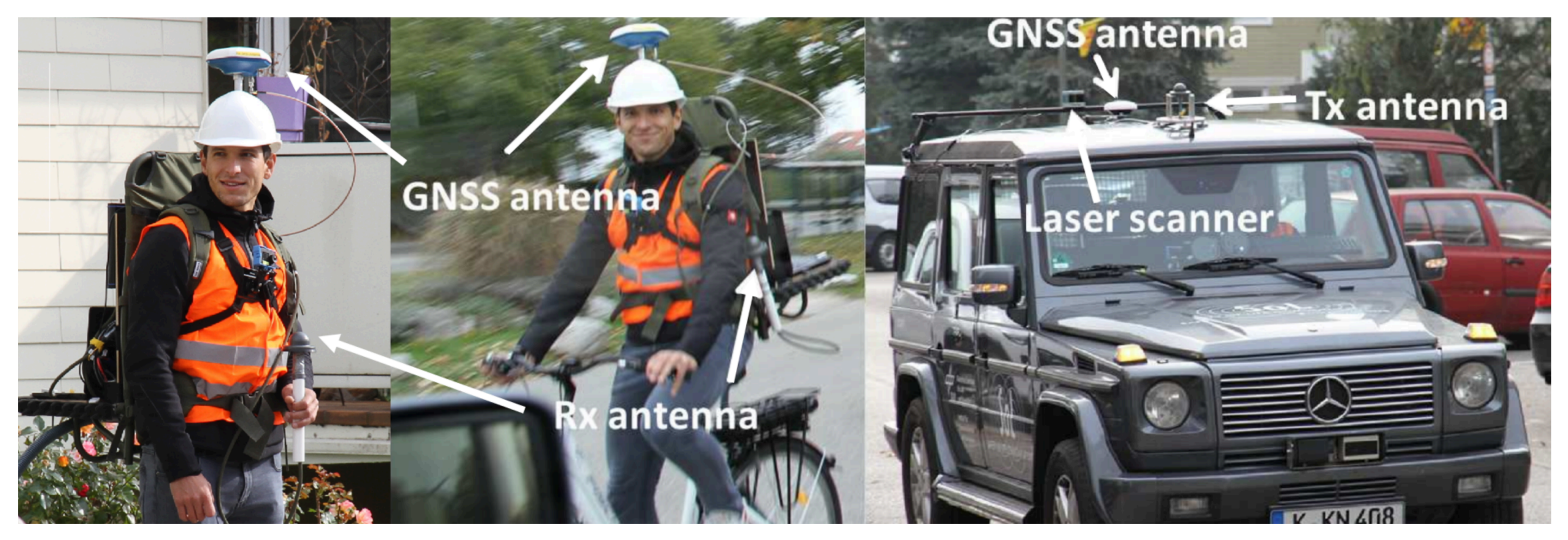
Antenna positions on the pedestrian, the cyclist, and the test vehicle.

The V2VRU measurement campaign was carried out using the RUSK-DLR channel sounder. The measurement signals were transmitted at a carrier frequency of $f_{c}=$ 5.2 GHz, which is close to the 5.9 GHz intelligent transport systems (ITS) band. The bandwidth was B= 120 MHz, thus providing a delay resolution of Δτ= 8.33 ns. During the measurements, the time-variant channel transfer function was recorded every $T_{g}=$ 1.024 ms, allowing a maximum Doppler shift of $f_{d\max}=$ 488 Hz to be resolved. The main measurement parameters can be found in Table 2. In order to record the position of the Tx and the Rx antennas, global navigation satellite system (GNSS) receivers were used. Two lidar sensors from Velodyne were mounted on the test vehicle and near the cyclist/pedestrian to provide a three-dimensional high-resolution point cloud of the environment. The antenna positions on the Tx and Rx sides can be seen in Figure 2. Note that a proper shift was used to match the position of the GNSS antenna to the position of the Tx and Rx antennas.

### 2.2. Measurement Scenarios

**Figure 3:**
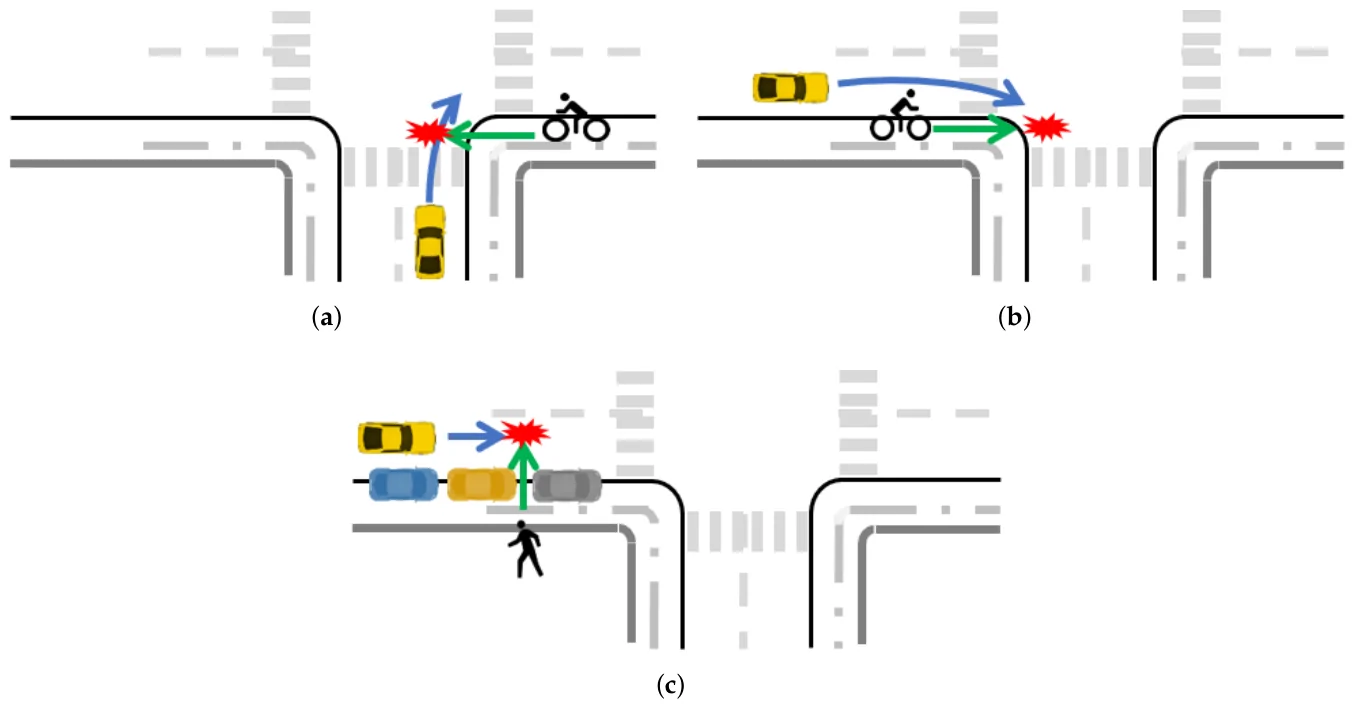
Illustrations of the accident scenarios in urban environment. The data collected in these scenarios are used to model the V2VRU channel. (**a**) Scenario 1. (**b**) Scenario 2. (**c**) Scenario 3.

Figure 1 displays the trajectories of the test vehicle, the cyclist, and the pedestrian on an aerial view for the accident scenarios. Three critical accident scenarios between vehicle and VRU were considered:

- Scenario 1 (Figure 3a): The vehicle is moving 100 m with an average velocity of 5–11 m s^−1^ towards the intersection, then turns right while the cyclist is moving 10 m with an average speed of 1.5 m s^−1^ toward the imaginary collision point from the right. This scenario accounts for 42% of total cyclist accidents as reported by the general association of the German insurance industry (GDV) [3]. In this scenario, the LoS between the vehicle and cyclist is initially blocked by buildings, then it becomes obstructed by parked vehicles as both the vehicle and cyclist approach the collision point. In this scenario, a total of 10 measurement runs were conducted.
- Scenario 2 (Figure 3b): In contrast to Scenario 1, the cyclist is moving parallel to the vehicle toward the collision point at the intersection. This scenario is less critical than Scenario 1 as it accounts for only 11% of total cyclist accidents [3]. In this scenario, the LoS between the vehicle and cyclist repeatedly transits between LoS and OLoS situations due to obstruction by parked vehicles. In this scenario, a total of 2 measurement runs were conducted. Although this number is limited, Scenario 2 can be considered exploratory. The cyclist moves parallel to the vehicle over a geometrically stable trajectory, which results in lower channel variability between runs than in Scenarios 1 and 3. The estimated LSP distributions show similar trends to those in Scenario 3, particularly in terms of K-factor and delay spread statistics, which supports the reliability of the collected data for parameterization purposes despite the limited number of runs.
- Scenario 3 (Figure 3c): The pedestrian is crossing the street with an average speed of 1 m s^−1^ while the vehicle is approaching from the left. In this scenario, the visibility between the vehicle and the pedestrian is partially or completely blocked by the parked vehicles. According to the national highway traffic safety administration (NHTSA) [2], this scenario is the most critical accident scenario for pedestrians and it accounts for 26% of pedestrian accidents. In this scenario, a total of 9 measurement runs were conducted.

The measurements were conducted at 5.2 GHz, which is adjacent to the 5.9 GHz ITS band used for V2X communication in Europe (ITS-G5) and North America (DSRC). The model parameters reported in this work are specific to this frequency band and to the measured urban street environment under the observed traffic and environmental conditions. The model should therefore be interpreted as a scenario-specific parameterization for this class of V2VRU situations rather than a general urban V2VRU model. Extending the characterization to other frequency bands, including sub-6 GHz cellular frequencies used in LTE/NR-V2X or future mmWave bands, as well as to other urban layouts and environmental conditions, is left for future work.

## 3. MPCs Parameters Estimation and Tracking

The behavior of the multipath channel can be described by the time variant channel impulse response (CIR).

A dynamic multipath estimator named the Kalman enhanced super resolution tracking (KEST), introduced in [30], is employed for estimating the parameters of the specular multipath components (SMCs). The estimated parameters are the absolute value of the amplitude, the delay, and the phase of each MPC at each time instant. The KEST algorithm uses the output of the space-alternating generalized expectation-maximization (SAGE) algorithm [31] as measurements within a Kalman filter. SAGE is used as a snapshot-based estimator that jointly estimates the complex amplitude and the delay for each MPC. Additionally, KEST consists of several Kalman filters in parallel using different model orders for estimating the number of MPCs. Based on the time-delay evolution structure of the MPCs, we found in [32] that the output of the MPCs parameters estimation not only contains SMCs but also contains diffuse multipath components (DMCs). For channel modeling, the dominant SMCs with long lifetimes need to be extracted and tracked over time.

A novel method to separate SMCs and DMCs was proposed in our previous work [33]. The method defines a search region around each MPC, and based on the number of neighboring MPCs within the search region, the examined MPC is classified as either SMC or DMC. Afterwards, the extracted SMCs need to be tracked over time. Therefore, in [33], we proposed a multipath component distance (MCD)-based tracking algorithm that measures the similarity between two MPCs based on their estimated delay and magnitude.

With the SMCs extracted and tracked, their parameters are employed to localize the physical scatterers in the propagation environment using a joint delay-Doppler estimation algorithm [34]. The estimated positions of the scatterers, presented in [33], are used to estimate the angle of departure (AoD) and angle of arrival (AoA) of the SMCs, which are required to later estimate the angular spreads (ASs).

## 4. Channel Model

In this section, we describe how the V2VRU channel model is developed. Based on the measured channels and the estimated MPCs parameters, the parameters of the channel model are first estimated. The model parameters are then used as an input to the channel simulator. Finally, the proposed channel model is validated by comparing the simulated channels with the measured channels.

### 4.1. Parameterization

All LSPs in this work are estimated using standard definitions and estimators consistent with WINNER II and 3GPP, which ensures reproducibility and allows direct comparison with existing channel models.

For developing a GSCM for V2VRU communication by adapting the WINNER II channel model, the LSPs need to be estimated. The LSPs describe the distribution of the power over the delay and angular dimensions, and control the evolution of the channel model. In this section, the LSPs are estimated in the power and delay domain, i.e., the shadow fading (SF), delay spread (DS), and KF, and in the angular domain, i.e., azimuth spread of departure (ASD) and azimuth spread of arrival (ASA). In order to maintain the spatial correlation of the LSPs observed in the measured channel, the autocorrelation of these LSPs is analyzed and the correlation distances are calculated. Furthermore, to ensure the spatial consistency, the cross-correlation coefficients among the LSPs are calculated. The model parameters can then be used as an input to the channel simulator. The first- and second-order statistics of the LSPs are estimated from the SMC parameters, i.e., the power, delay, and angles, that were previously estimated from the measurement data and from scatterer locations.

The log-normal distribution is widely used to model the LSPs in models like WINNER II, 3GPP, and Quadriga. Following the WINNER II approach, the LSPs are fitted to log-normal distributions, ensuring compatibility with standards and comparability with existing literature. Root mean square error (RMSE) is used to quantify the root average squared difference between the empirical and theoretical CDFs as in [35,36,37], providing an evaluation of the fit. The RMSE also enables straightforward evaluation of the similarity between the CDFs of LSPs derived from measured and simulated channels, reinforcing the validity of the model.

Due to the non-stationarity of the channel, the channel is divided into regions, within which the wide-sense stationary (WSS) assumption holds. The LSPs and their correlations are evaluated within these regions using a sliding average window of length of 15λ (0.86 m). The length of the window, i.e., the stationarity distance, is found to be valid for all scenarios based on the estimated stationarity distance in our previous work [38].

The KF and DS are found to vary significantly when the propagation condition changes, which makes it necessary to treat them separately for different propagation states. Specifically, the KF is only physically meaningful when a dominant LoS or near-LoS component is present, while the DS increases markedly when the LoS is fully blocked. For this reason, the dataset is divided into two parts: the LoS or OLoS part, where the link has an unobstructed or partially obstructed direct path, and the NLoS part, where the LoS is fully blocked by buildings or parked vehicles. The classification is based on the geometric visibility between Tx and Rx, determined from the GNSS trajectory data and the lidar-based point cloud. The appearance of the LoS starts at an average Tx–Rx distance of 15 m in Scenario 1, and at 9 m in both Scenario 2 and Scenario 3. Unlike the KF and DS, the SF, ASD, and ASA are estimated for the whole experiment with no distinction between LoS and OLoS/NLoS situations.

#### 4.1.1. Path Loss

Path loss models for the three scenarios are proposed. The path loss is calculated from the measured channels, and it includes the power contribution of both the SMCs and the DMCs. The path loss does not only provide valuable insight into the impact of the propagation environment on the received power, but it is also used as an input to the channel model.

The path loss can be expressed in logarithmic scale as a sum of two components, namely, the distance-dependent path loss model and the SF:

(1) $$P_{L}=\underset{\mathrm{distance}\text{-}\mathrm{dependent}\;\mathrm{path}\;\mathrm{loss}\;\mathrm{model}}{\underset{︸}{\bar{P_{L}\left(d\right)}}}+\underset{\mathrm{shadow}\;\mathrm{fading}}{\underset{︸}{X}}.$$

The SF is caused by shadowing processes and leads to a change in the local mean of the path loss over relatively large distances, whereas the small-scale fading is the variation due to the superposition of multiple propagation paths. It leads to power variations when moving over relatively short distances in the order of the signal wavelength. The small-scale fading is extracted by applying a sliding window with length of 10λ.

The log-distance path loss model is used to predict the propagation loss in different environments. The average large-scale path loss is a function of the separation distance between the Tx and Rx as expressed by

(2) $$\bar{P_{L}\left(d\right)}=P_{L}\left(d_{0}\right)+10n\log_{10}\left(d/d_{0}\right),$$

where $\bar{P_{L}\left(d\right)}$ denotes the path loss in dB at a distance *d* between Tx and Rx, $P_{L}\left(d_{0}\right)$ is the path loss at a reference distance $d_{0}$, and *n* is the path loss exponent. Using linear regression analysis, the path loss exponent *n* that minimizes the difference between the measured and modeled values is determined. In scenarios where the LoS becomes partially or completely obstructed, it has been found that the measured path loss can be more accurately represented by a dual-slope log-distance path loss model. The dual-slope model is given by (3), where $d_{b}$ is the breakpoint distance at which the second slope begins. $n_{1}$ and $n_{2}$ are the path loss exponents of the first and second slope, respectively, and they provide an indication of how the signal attenuates with the Tx–Rx separation distance.

(3) $$\bar{P_{L}\left(d\right)}=\left\{\begin{array}{c} P_{L}\left(d_{0}\right)+10n_{1}\log_{10}\left(d/d_{0}\right),\;\mathrm{if}\;d\leq d_{b}\\ P_{L}\left(d_{0}\right)+10n_{1}\log_{10}\left(d_{b}/d_{0}\right)+10n_{2}\log_{10}\left(d/d_{b}\right),\;\mathrm{if}\;d>d_{b}\;. \end{array}\right.$$

The breakpoint distance $d_{b}$ is not treated as a free fitting parameter. It corresponds physically to the Tx–Rx separation at which the propagation condition changes, from OLoS to NLoS in Scenario 1, and from LoS to OLoS in Scenarios 2 and 3, and is identified directly from the GNSS-recorded trajectories combined with the lidar-based point cloud of the environment. Given $d_{b}$, the path loss exponents $n_{1}$ and $n_{2}$ are estimated independently for each distance range using linear regression, i.e., by minimizing the sum of squared residuals between the measured and modeled path loss values.

Note that $d_{b}$ here is distinct from the LSP correlation distance $d_{c}$ introduced later in this section.

#### 4.1.2. Shadow Fading

As defined in (1), the SF is the zero-mean residual obtained after subtracting the fitted distance-dependent path loss model from the measured path loss. The SF is usually modeled as a normally-distributed random variable with zero mean. In all three scenarios, the SF is estimated for the whole experiment, i.e., the data set is not divided based on the propagation condition.

#### 4.1.3. Delay Spread

The DS is widely used to statistically describe delay characteristics of wireless channels. Due to the multipath propagation, the Rx receives several copies of the transmitted signal. Each copy arrives from different direction with different gain and delay. The delay dispersion of the propagation paths is described by the DS metric. The DS in the CIR snapshot at time *t* is calculated from the estimated parameters of the SMCs according to [39]

(4) $$\sigma_{\tau }=\sqrt{\frac{\sum_{i=0}^{N(t)-1}P_{i}\tau_{i}^{2}}{\sum_{i=0}^{N(t)-1}P_{i}}-{\bar{\tau }}^{2}},$$

where $\bar{\tau }$ is the mean excess delay calculated as in [39]

(5) $$\bar{\tau }=\frac{\sum_{i=0}^{N(t)-1}P_{i}\tau_{i}}{\sum_{i=0}^{N(t)-1}P_{i}},$$

and where $P_{i}$ and $\tau_{i}$ are the power and delay of the *i*-th MPC, and N(t) is the number of MPCs at time *t*. The DS is calculated for each scenario from all measurement runs and then, following the WINNER II approach, fitted to a log-normal distribution with a unit of $\log_{10}\left(s\right)$.

#### 4.1.4. K-Factor

The KF is an important parameter that provides an indication of the energy proportion of the MPCs and hence quantifies the multipath richness of the communication channel. The KF is defined as the ratio of the energy of the dominant component that consists of the LoS component and the unresolved ground reflection to the energy of all other components. In the literature, several approaches are proposed to estimate the KF. A widely used technique to estimate the KF from the measured CIR is introduced in [40] based on the method of moments (MoM). However, as stated earlier, the narrowband KF in this work is calculated for each measurement snapshot from the estimated parameters of the SMCs and it is calculated according to [39]

(6) $$K=\frac{P_{\mathrm{LoS}}}{P-P_{\mathrm{LoS}}}\;\;,$$

where $P_{\mathrm{LoS}}$ is the power of the LoS path, and *P* is the total power. Typically the KF is in dB and it is modeled by a log-normal distribution.

It should be noted that the MoM estimator proposed in [40] is intended for narrowband envelope measurements. Since the data in this work consists of MPC parameters extracted from wideband channel sounding, computing the KF directly from the power ratio in (6) is the more appropriate approach for this data type.

#### 4.1.5. Angular Spreads

The AS is the parameter that describes the dispersion of propagation paths in the angular domain, i.e., it measures how the multipath power is spread out in the spatial domain. The AS is calculated utilizing the path angles, i.e., AoD and AoA, and the path powers. The AoD and AoA are calculated from the scatterer locations. The reference angle, i.e., the angle 0°, is set to be the angle between the Tx and the Rx. The ASD and ASA are calculated in each measurement snapshot as in [41]

(7) $$\sigma_{\phi }=\sqrt{\frac{\sum_{i=0}^{N(t)-1}{\left(\xi \left(\phi_{i}-\bar{\phi }\right)\right)}^{2}P_{i}}{\sum_{i=0}^{N(t)-1}P_{i}}}\;,$$

where $P_{i}$ and $\phi_{i}$ are the power and angle of the *i*-th MPC, function $\xi \left(.\right)$ maps angles to (−180, 180) degrees, and the power weighted mean angle $\bar{\phi }$ is given by

(8) $$\bar{\phi }=\frac{\sum_{i=0}^{N(t)-1}\xi \left(\phi_{i}\right)P_{i}}{\sum_{i=0}^{N(t)-1}P_{i}}\;.$$

The ASD and the ASA are estimated for the whole experiment because, unlike the DS and KF, the difference in angle spread in different propagation cases, i.e., LoS, OLoS, and NLoS, was found to be insignificant.

#### 4.1.6. LSP Correlations

In order to maintain the spatial correlation of the LSPs observed in the measured channel, the autocorrelation of these LSPs is analyzed and the correlation distances are calculated. Furthermore, to ensure the spatial consistency, the cross-correlation coefficients among the LSPs are calculated.

The correlation distance $d_{c}$ of an LSP indicates how long the channel can be assumed stationary for this specific LSP. The correlation distance is used as a model parameter to maintain the spatial correlation of the LSP observed in the measured channel. It is employed in generating the correlated 2-dimensional (2D) map of the LSP by determining the length of the channel segment, or the length of the traveled distance, in these maps. The correlation distance is calculated from the autocorrelation function of the evaluated LSP as follows

(9) $$r\left(\Delta d\right)=E\left[x(d)x(d+\Delta d)\right],$$

where $E\left[.\right]$ denotes the expected value, *d* is the distance between Tx and Rx, Δd is the change in the Tx–Rx distance, and *x* is the LSP under evaluation in log domain. The correlation distance $d_{c}$ is defined as the value of Δd at which the value of the normalized autocorrelation function drops to $e^{-1}$. Note that, in case the normalized autocorrelation function has a value of $e^{-1}$ at different values of Δd, the shortest Δd is the correlation distance.

The normalized autocorrelation function is obtained as $\hat{r}\left(\Delta d\right)=r\left(\Delta d\right)/r\left(0\right)$, where r(0) is the value of the autocorrelation function at zero lag, such that $\hat{r}\left(0\right)=1$.

In order to ensure spatial consistency of the channel, the cross-correlations between all LSP pairs are needed. This metric describes the linear dependency between the LSPs. The cross-correlation coefficient is given by

(10) $$\rho_{x,y}=\frac{\sum_{m=1}^{M}\left(x\left(m\right)-\bar{x}\right)\left(y\left(m\right)-\bar{y}\right)}{\sqrt{\sum_{m=1}^{M}{\left(x\left(m\right)-\bar{x}\right)}^{2}\sum_{m=1}^{M}{\left(y\left(m\right)-\bar{y}\right)}^{2}}}\;\;,$$

where *x* and *y* are the measured sequences of the two LSP pairs with length *M*, and $\bar{x}$ and $\bar{y}$ are the sample means of *x* and *y*, respectively.

Cross-correlation coefficients take values from −1 to 1. A negative coefficient indicates a decrease in parameter *x* if parameter *y* increases and vice versa. Positive coefficient means that *x* and *y* increase and decrease simultaneously. When the coefficient is zero there is no dependency between the two parameters.

### 4.2. Implementation

The modeling approach in this work follows the QuaDRiGa modeling approach [42]. The QuaDRiGa model extends the well known WINNER II [14] and WINNER+ [43] models by adding time evolution and 3D propagation. In this model, the trajectories of both the Tx and the Rx are divided into segments. A channel segment can be seen as a part of the Tx and the Rx routes, in which the LSPs do not change considerably. The positions of the scatterers are controlled by the angular and delay spreads. Within a channel segment, the scatterer positions are fixed. However, due to the movement of the Tx and the Rx within the segment, the propagation path parameters, i.e, the power, delay, phase, and angles, will change. The time evolution of the channel due to the movement of the Tx and Rx is accomplished by the so-called drifting and the birth-death process, i.e., appearance and disappearance of the scatterers. Drifting, on the one hand, deals with the time evolution inside a channel segment. It was introduced in the Spatial Channel Model Extended (SCM-E) [44]. When the Tx and the Rx move from one channel segment to another, different scatterers will be created. The birth and death process of the scatterers, on the other hand, ensures a smooth transition between adjacent channel segments.

**Figure 4:**
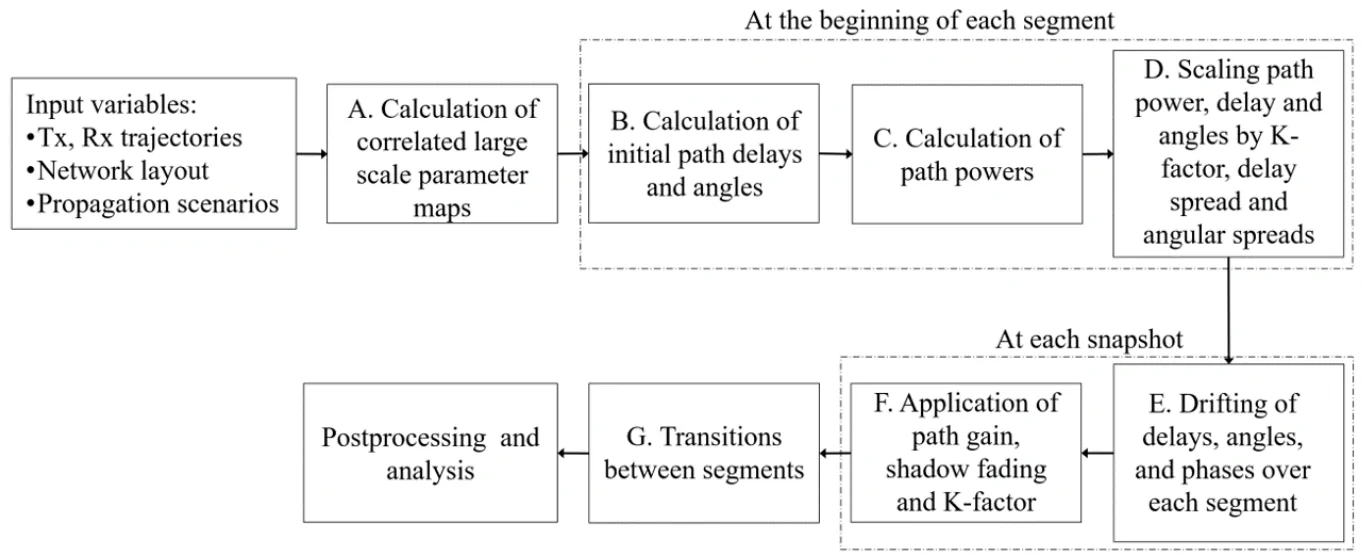
Steps for calculating of time-evolving channel coefficients.

The block diagram in Figure 4 shows the procedure of the channel generation. In the first step, the user provides the trajectories of the Tx and Rx and the propagation scenario. The network layout is created by defining the number of the Tx and Rx and antenna configurations. A detailed description of the channel coefficient generation steps is found in [14,42].

In addition to the LSPs, the scaling coefficients, also called the proportionality factors, are required as an input to the channel model. They are used to scale the path delays and angles to ensure that the differences in the spreads are reflected in the path powers (Step D in Figure 4). The scaling coefficient of the delay $g^{\mathrm{DS}}$ is defined as the ratio between the standard deviation of the path delays and the root mean square (rms) DS. Similarly, the scaling coefficients of the angles, $g^{\mathrm{ASD}}$ and $g^{\mathrm{ASA}}$, are defined as the ratio between the standard deviation of the path angles and the AS [14]. Table 3 summarizes the mean values of the scaling coefficients estimated from the measurement data in all scenarios.

Based on the measurement data, the number of MPCs used in the channel simulations is set to 8 for all scenarios. Changing the number of MPCs was found to have an insignificant impact on the simulated channels.

### 4.3. Validation

The comparison between simulated and measured channel statistics serves two purposes that are worth distinguishing. The first is to verify that the QuaDRiGa implementation correctly translates the LSP inputs into channel realizations with matching statistics. The second, and more directly relevant to this work, is to confirm that the estimated LSPs are representative of the measured V2VRU propagation environment. If the simulator, driven by the proposed model parameters, reproduces the measured channel statistics, this provides evidence that the estimation procedure has captured the essential propagation characteristics of the V2VRU channel. The link-level results in Section 5.7 then serve as an independent application-level check.

The GSCM for V2VRU communications is validated by comparing the simulated channels with the measured channels. The simulated channels are generated by the WINNER-type QuaDRiGa simulator [42]. The model parameters are extracted from the measured channels and used as an input to the channel simulator. The channel model is validated by comparing the distributions of the model parameters, extracted from the simulated channels, with their counterparts extracted from the measured channels which are referred to as the model input. The similarity between the CDFs of LSPs derived from measured and simulated channels are evaluated using the RMSE metric. The validation is performed using a group of 100 simulated channels produced by 100 simulation runs for each scenario. These simulation runs are carried out using Tx and Rx routes similar to the routes during the channel measurements. Therefore, the resulting simulated channel in each run has a similar number of snapshots as the measured channel. Furthermore, the channels are generated for a center frequency of 5.2 GHz and a bandwidth of 120 MHz. It must be also noted that only SMCs are considered when estimating the LSPs and generating the simulated channels. As for the DMCs, this model incorporates only their power contributions in the proposed path loss models.

## 5. Results

Following the channel modeling approach introduced in the previous section, this section presents results of estimating and modeling all model parameters. The proposed channel models are then validated by comparing the distributions of the model parameters, extracted from the simulated channels, with their counterparts extracted from the measured channels. Table 4, Table 5, Table 6 and Table 7 summarize the comparison results for each scenario. Moreover, the impact of the channel model on the performance of the communication system is evaluated through link-level simulations.

### 5.1. Path Loss

**Figure 5:**
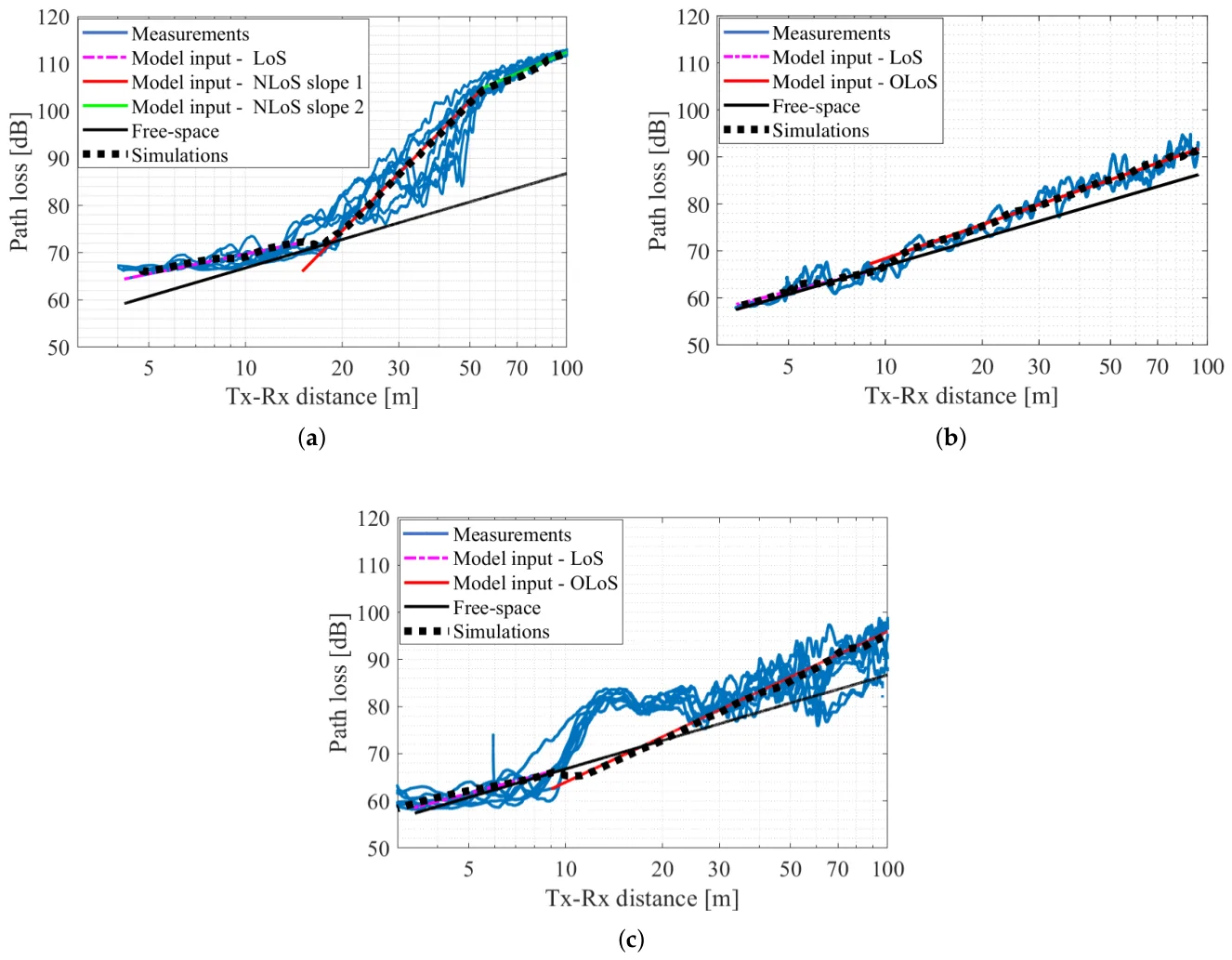
Comparison of the path loss variations for the three scenarios. (**a**) Scenario 1. (**b**) Scenario 2. (**c**) Scenario 3.

Figure 5 shows the measured path loss variations with the Tx–Rx distance and the proposed path loss models for the measured scenarios in the urban environment. Note that the plotted measured path loss is for a total of 10 measurement runs in Scenario 1, 2 runs in scenario 2, and 9 runs in scenario 3. Table 4 summarizes the results of fitting the measured path loss linearly to the proposed models.

It should be noted that the value of $P_{L}\left(d_{0}\right)=-15.16$ dB reported for Scenario 1 OLoS/NLoS Slope 1 is a result of extrapolating the fitted model back to the reference distance $d_{0}=\;$ 1 m. This value does not represent a physically measured path loss at that distance, as the model is only valid within the fitted distance range of 15–100 m.

The standard deviation σ in Table 4 reflects the spread of the shadow fading residuals around the fitted path loss model. In Scenario 1, σ is not listed separately for the OLoS/NLoS Slope 1 segment because the shadow fading is estimated over the complete dataset without separating propagation conditions, as explained in Section 4. The single reported value therefore applies to the combined OLoS/NLoS distance range.

In Scenario 1 (Figure 5a), due to movement of the vehicle (Tx) and the cyclist (Rx), the Tx–Rx link transits between NLoS, OLoS, and LoS. A NLoS situation arises due the blockage of the LoS by buildings between 32 m and 100 m. Due to the obstruction, an extra loss of maximum 25 dB is experienced when compared to the free space path loss. Between 15 m and 32 m the LoS becomes obstructed by the parked vehicles which causes a diffraction loss of 5–15 dB. A LoS state appears only shortly before the collision between the vehicle and the cyclist at the intersection. When compared to the free space path loss, a difference up to 7 dB can be noticed. This difference is partially due to the ground and self-body reflection. The measured path loss is found to be more accurately represented by a dual-slope log-distance path loss model given by (3) for distances between 15 m and 100 m with a breaking distance of 55 m. Path loss exponents of 6.9 and 2.9 for the first and the second slopes, respectively, are estimated. The path loss during LoS is modeled by a single-slope log-distance model with an estimated path loss exponent of 1.4.

In Figure 5b the path loss for Scenario 2 is modeled by two single-slopes log-distance models. The first slope is for distances up to 9 m and covers the LoS area prior to the collision with an estimated path loss exponent of 1.7. For distances between 9 m and 100 m, the link between the vehicle and the cyclist becomes partially obstructed by parked vehicles which causes a diffraction loss of maximum 5 dB. The estimated path loss exponent equals 2.4, which is slightly above the one of the free space model.

In Scenario 3 (Figure 5c) the estimated path loss exponent for the LoS area, i.e., up to 9 m, does not vary much from Scenario 2 with an exponent of 1.8. However, the path loss experiences a sudden increase by 15 dB at around a Tx–Rx distance of 10 m due to the obstruction of the LoS by a large vehicle. The path loss between 9 m and 100 m shows rapid variations in the order of 5 to 15 dB due to obstruction of the LoS by parked vehicles with an estimated path loss exponent of 3.2.

Two features of the proposed path loss models in Figure 5 require clarification. In Figure 5a, the model curve shows a drop at $d_{b}$, where the OLoS and NLoS slopes meet. This drop is not present in the measured data. It occurs because the two slopes are fitted independently, so the NLoS slope is free to start at a different value than where the OLoS slope ends. Imposing a continuity condition at $d_{b}$ was tested but led to a noticeably worse fit on both sides and was therefore not applied. In Figure 5c, the model deviates from the measurements in the 10–20 m range. Here, the measured path loss increases abruptly over a very short distance due to obstruction by a single large parked vehicle, which the linear model cannot follow exactly.

In order to validate the proposed channel model in terms of the path loss, the local mean path loss, calculated from all 100 simulated channels and depicted by the dashed black curve, is compared with the proposed path loss model. It can be seen that the V2VRU channel model clearly provides an almost perfect match in terms of path loss in all three measured scenarios.

The LoS appearance distances stated in Section 4 are average values across measurement runs. The actual appearance distance varies between runs depending on the exact vehicle and VRU positions, which explains why some individual path loss traces in Figure 5 show the LoS transition at a slightly different distance than what the model assumes.

### 5.2. Shadow Fading

**Figure 6:**
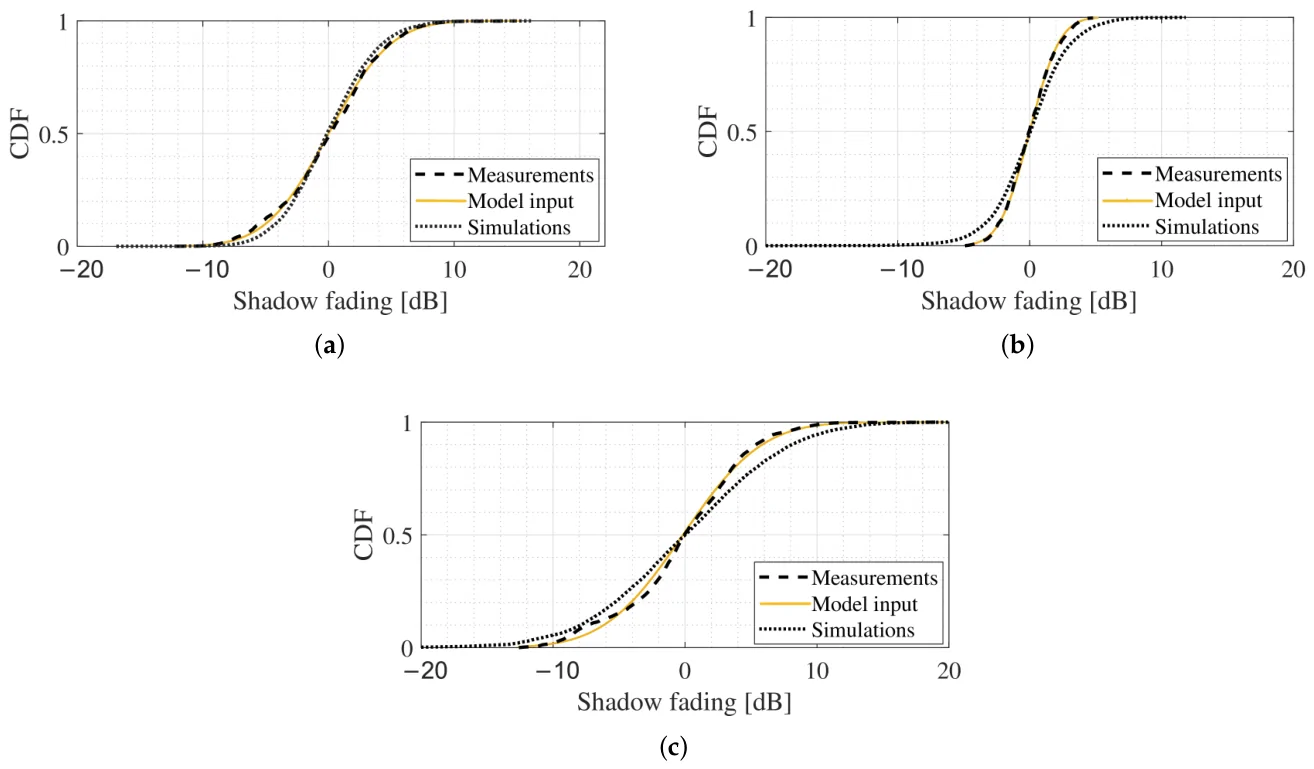
Comparison of the distribution of the shadow fading (SF) for the three scenarios. (**a**) Scenario 1. (**b**) Scenario 2. (**c**) Scenario 3.

The parameterization and validation of the channel model in terms of SF for each scenario are shown in Figure 6. Positive SF values indicate a higher overall path loss, while negative SF values mean smaller overall path loss. With an RMSE of 0.012, Scenario 3 shows slight differences between the measured SF’s CDF and the log-normal fit. Despite this, the log-normal distribution provides a good approximation of the measured SF distribution, especially in Scenarios 1 and 2, with RMSE values of 0.007 and 0.006, respectively. The dotted black curve represents the CDF of the SF derived from 100 simulated channels. In Scenario 1, a good alignment is noticed between the simulated SF and the model input, i.e., the log-normal fit, with standard deviation values of 3.20 dB for the model input and 3.35 dB for the simulated channels. A minor mismatch is observed in Scenario 2, where the standard deviation from the simulated channels is around 1 dB larger than that of the model input. A similar mismatch can also be seen in Scenario 3, where the simulated channels have higher SF with a standard deviation of 6.31 dB compared to 4.69 dB for the model input. These observations are quantified by the RMSE values. In Scenario 1, the small RMSE of 0.015 between the measured and simulated CDFs indicates a strong agreement. However, in Scenarios 2 and 3, the mismatches result in higher RMSE values of 0.021 and 0.031, respectively.

### 5.3. Delay Spread

**Figure 7:**
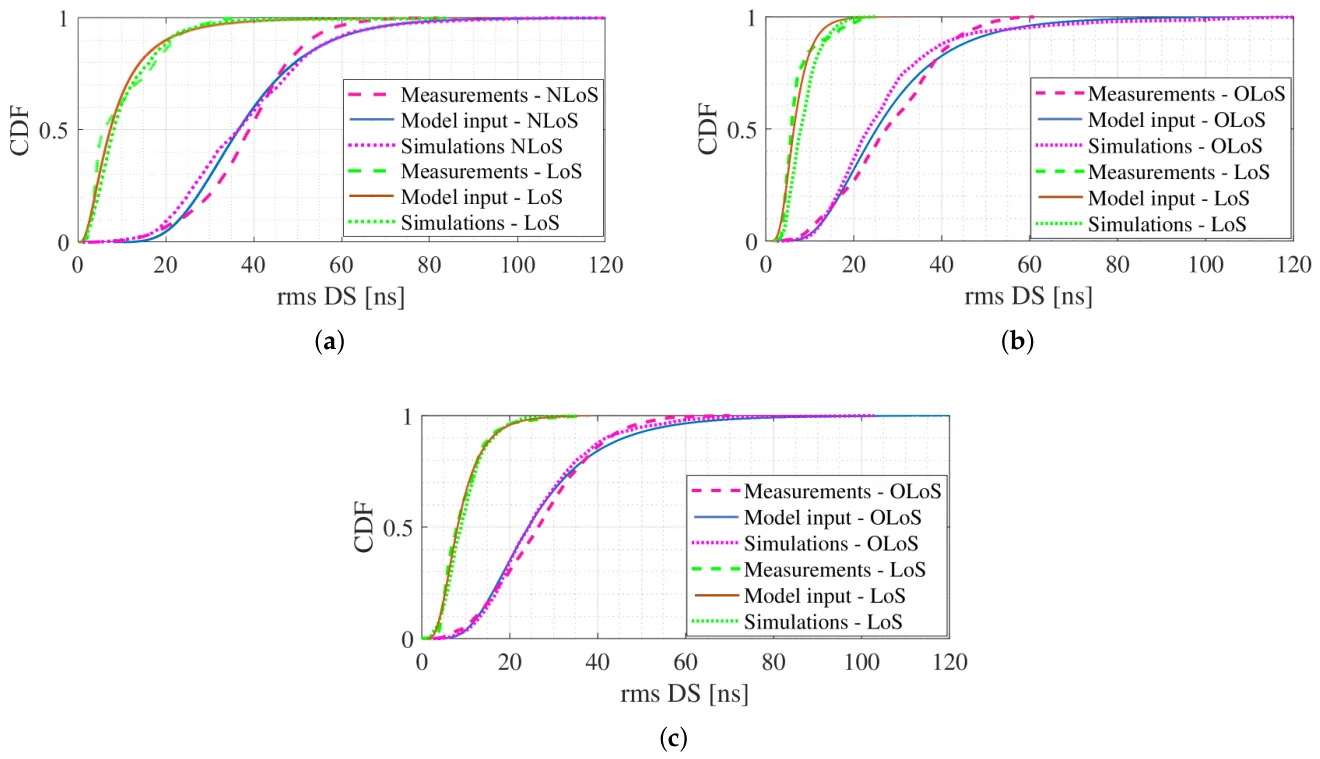
Comparison of the distribution of the root mean square delay spread (rms DS) for the three scenarios. (**a**) Scenario 1. (**b**) Scenario 2. (**c**) Scenario 3.

Figure 7 illustrates the rms DS results across different scenarios, each divided into two according to the propagation conditions: LoS and OLoS/NLoS. The log-normal fit (model input) provides a good approximation of the measured CDFs in all scenarios and propagation conditions. The RMSE values are between 0.004 and 0.032 which indicates a good fit. However, the log-normal fit and the simulated CDFs have slightly larger DS values compared to the measured channels. In Scenario 1, the measured channels have mean DS values of 7.41 ns for LoS and 36.31 ns for NLoS. The simulated mean DS values are 8.32 ns and 34.67 ns for LoS and NLoS, respectively. Similar agreement is observed in Scenarios 2 and 3, as detailed in Table 5, Table 6 and Table 7. While the distribution of the rms DS from the simulated channels has a good alignment with the measured one in most cases, a mismatch occurs in Scenario 2 LoS, where the simulated CDF differs from the measured one, resulting in an RMSE of 0.09. In all other scenarios and conditions, the RMSE between the measured and simulated CDFs remains at or below 0.036.

The slightly larger DS values in the simulated channels can be attributed to two factors. First, the stochastic scatterer placement in QuaDRiGa smooths out short-term variations in the delay and power of weak MPCs, which leads to a positive bias in the simulated DS. Second, weak MPCs near the noise floor are difficult to estimate reliably, which likely causes a slight underestimation of the DS in the measured channels. Both effects shift the results in the same direction and together explain the observed positive offset.

### 5.4. K-Factor

**Figure 8:**
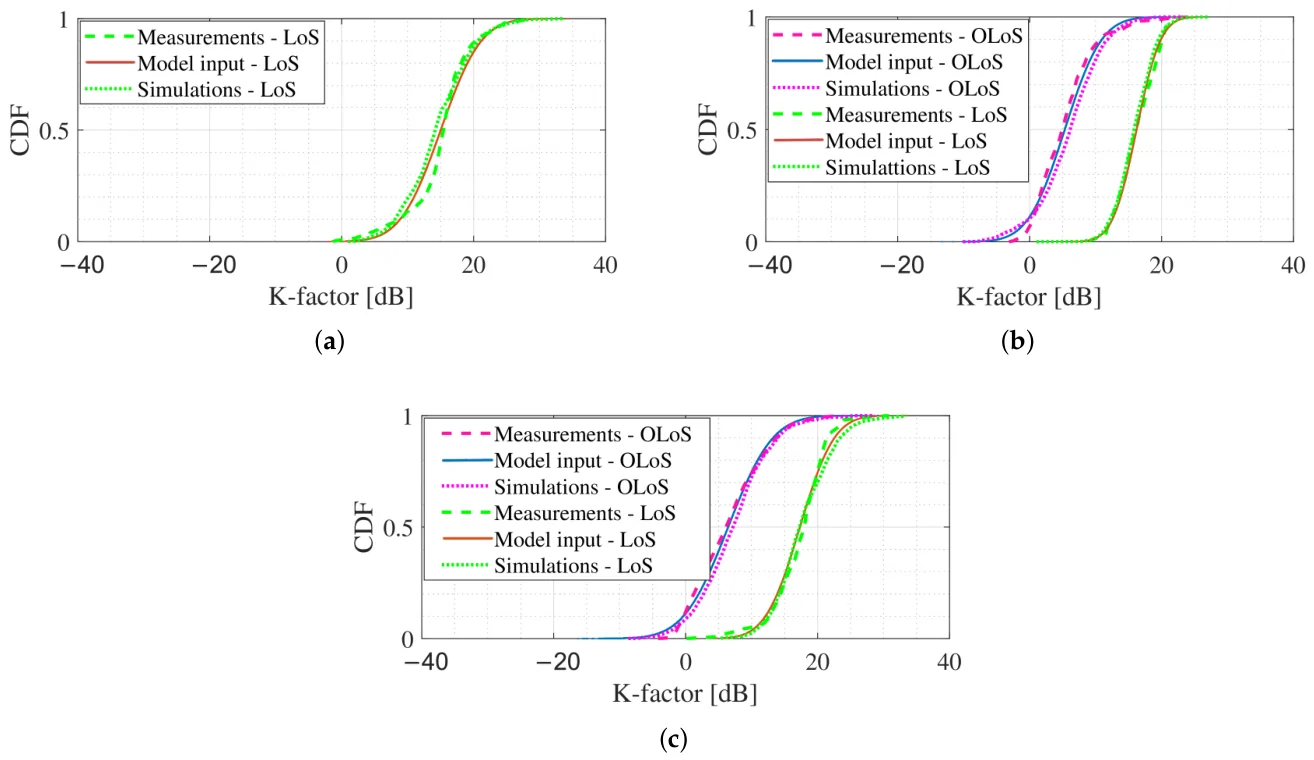
Comparison of the distribution of the K-factor (KF) for the three scenarios. (**a**) Scenario 1. (**b**) Scenario 2. (**c**) Scenario 3.

Figure 8 illustrates the CDFs of the measured KF, the log-normal fit (model input), and the simulated channels for the three scenarios. The larger KF observed in the LoS condition compared to OLoS is attributed to the presence of a strong, unobstructed LoS path. In the OLoS condition, the LoS path is partially or completely blocked by parked vehicles, which results in attenuation due to diffraction loss, as discussed in [45]. The parametrization results show that the log-normal distribution provides a good fit to the measured KF in all scenarios with relatively small RMSE values between 0.012 and 0.024. In Scenario 1, the mean and standard deviation of the KF in the model input are 15.02 dB and 4.81 dB, respectively. The simulated channels closely match the KF in the measured data with a mean of 14.44 dB and a standard deviation of 4.98 dB, and with an RMSE of 0.031. Similarly, in Scenarios 2 and 3, the simulated KF values align well with the model input in both LoS and OLoS. The RMSE between the measured and simulated CDFs in all scenarios is at or below 0.038, which indicates that the proposed model captures the KF characteristics in different propagation conditions.

### 5.5. Angular Spreads

**Figure 9:**
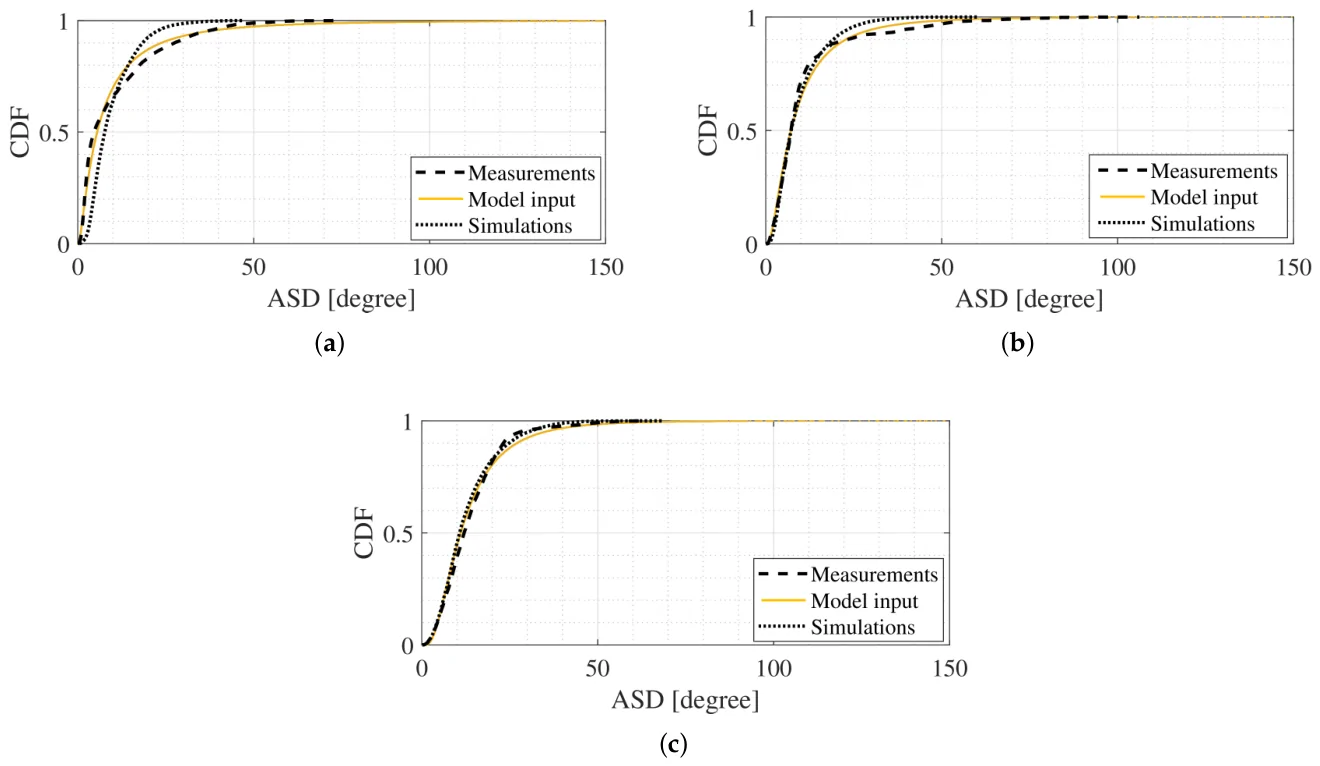
Comparison of the distribution of the azimuth spread of departure (ASD) for the three scenarios. (**a**) Scenario 1. (**b**) Scenario 2. (**c**) Scenario 3.

Figure 9 compares the CDFs of the measured ASD, its log-normal fit (model input), and the simulated channels across the three scenarios. The parametrization results demonstrate that the measured ASD closely follows the log-normal fit in all scenarios, with minor mismatches and a maximum RMSE of 0.017. The validation results show that the ASD of the simulated channels has a good alignment with the measured ASD in Scenarios 2 and 3 in terms of both the mean and the standard deviation. However, in Scenario 1, the simulated channels produce a larger ASD, with a mean of 7.76° compared to 5.50° in the model input. This mismatch results in an RMSE of 0.097, which is higher than the RMSE in the other scenarios. The mismatch in Scenario 1 can be related to challenges in estimating the MPCs angles in NLoS conditions. The estimation of scatterer locations relies on the delay and Doppler information of the MPCs, which, together with the transmitter and receiver positions, are used to derive the AoD and AoA. During NLoS in Scenario 1, the MPCs have short lifetimes and weak power levels which lead to significant error in phase estimation. These errors reduce the reliability of scatterer location estimations and can thus reduce the accuracy of the calculated path angles and angular spreads, which explains the mismatch.

**Figure 10:**
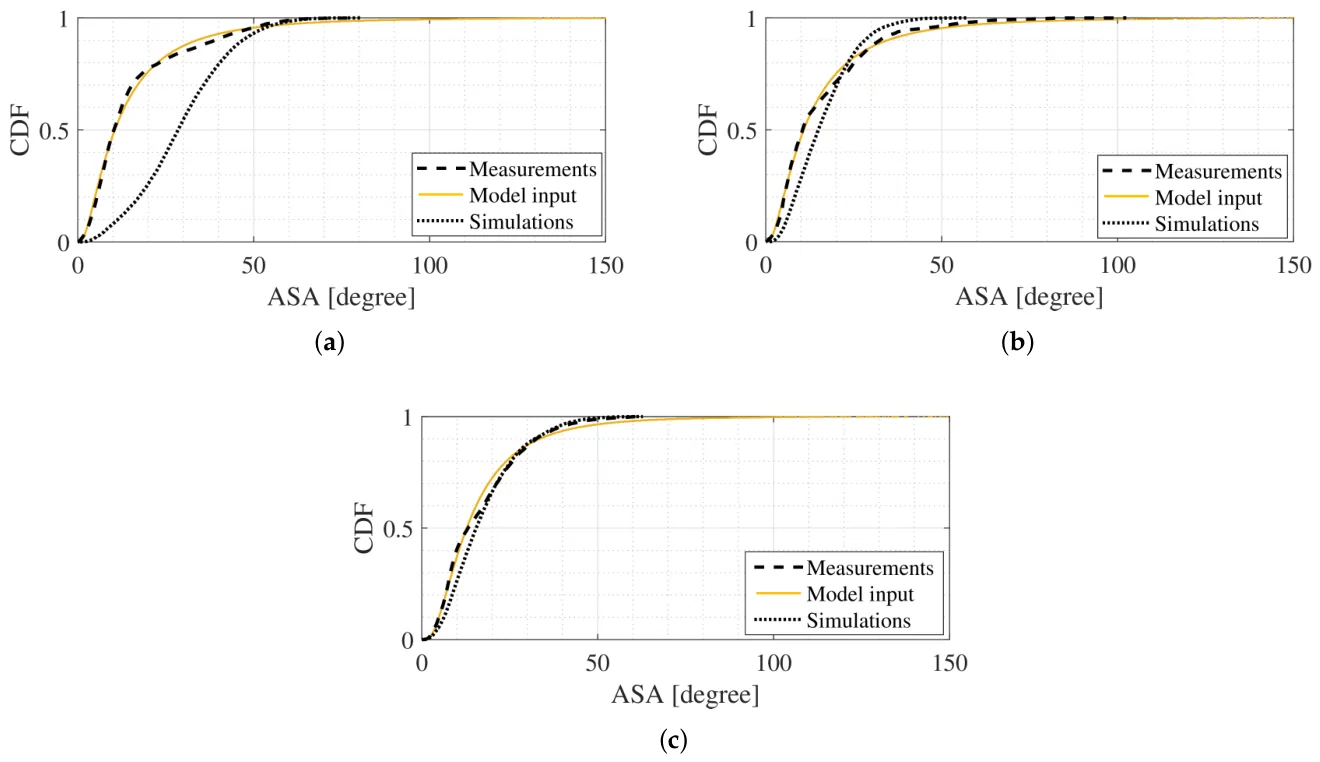
Comparison of the distribution of the azimuth spread of arrival (ASA) for the three scenarios. (**a**) Scenario 1. (**b**) Scenario 2. (**c**) Scenario 3.

The results for the ASA are shown in Figure 10. The parametrization results show that the measured ASA follows the log-normal fit (model input) in all three scenarios with some minor mismatches and a maximum RMSE of 0.011. In Scenario 1, the model input gives a mean ASA of 10.47° which is significantly smaller than the mean of 25.15° observed in the simulated channels, resulting in a high RMSE of 0.159. The simulated channels in Scenario 2 and Scenario 3 show a much better match with the measured ASA. In these scenarios, the simulated channels follow the model input more closely and have better agreement in the mean and the standard deviation. However, in Scenario 1, the mismatch between the model input and the simulated channels is noticeable. This mismatch is probably due to the limitations of the scattering model used in the channel model implementation. The channel generation procedure includes both single- and double-bounce models, but due to the SISO measurement limitation, only a single-bounce model is considered during the parametrization and only the last-bounce scatterers are localized. This simplification contributes to the observed deviations in the angular spread. The mismatch is most relevant for applications that rely on spatial channel information, such as beamforming and angle-based localization, and addressing it requires MIMO measurements capable of resolving multi-bounce contributions, which is left for future work.

### 5.6. LSP Correlations

The correlation distance is calculated from the autocorrelation function of the evaluated LSP and used as a model input to simulate channels that maintain the spatial correlation observed in the measured channels. It must be noted that when the 2D correlated maps of the LSPs are generated during channel simulation, the cross-correlations between LSP pairs are applied to the correlated maps (Step A in the channel coefficient generation in Figure 4). Therefore, correlation distances of LSPs are distorted and not independent from each other anymore.

As can be seen from Table 5, Table 6 and Table 7, on the one hand, the KF, DS, ASD, and ASA show relatively high spatial correlation which implies a slow change in their values over time in all scenarios. On the other hand, the SF experiences less spatial correlation which can be explained by the intermittent blockage of the LoS by parked vehicles. The results show that the correlation distances of the KF and DS during the LoS part are much shorter than in the NLoS/OLoS part. This is attributed to the fact that the length of the LoS part, within which the autocorrelation function is evaluated, is limited to only few meters prior to the collision point, i.e., less than 10 m in Scenario 1 and less than 5 m in Scenario 2 and Scenario 3. It can be noticed that the simulated channels in all scenarios show an increase in the correlation distances of most LSPs. This mismatch between the model input and the simulated channels in terms of correlation distance is inevitable due to the aforementioned cross-correlation impact. However, when an LSP shows an insignificant correlation with another LSP, the change in its correlation distance will be small. For example, as summarized in Table 8, in Scenario 3, the ASD has almost no correlation with the other LSPs in the model input except with the ASA. As a result, the correlation distance calculated from the simulated channels has an almost perfect agreement with the model input.

Table 8 summarizes the mean cross-correlation values of LSP pairs that were set to the model input in comparison with the values that are calculated from the simulated channels for all scenarios. Note that all cross-correlation matrices are positive definite. There are 5 different LSPs which result in 10 cross-correlation values. For each scenario, 10 values are obtained from the measurements and 10 values from the simulated channels. The cross-correlation values measure the inter-dependency between each pair of the LSPs. Note that no distinction between different propagation conditions is made for the calculation of the cross-correlation, i.e., the complete data set of each measurement run is considered.

In all scenarios, the cross-correlations between the SF, KF, and DS calculated from the simulated channels are in good agreement with the model input. The SF is found to be positively correlated with the KF while negatively correlated with the DS. The ASD shows weak or no dependency on the SF, KF and DS. Similarly, the ASA shows no correlation with the KF and DS in all scenarios, while it has a weak dependency on the SF in Scenarios 2 and 3. In all scenarios, both ASD and ASA are negatively correlated with the SF and KF and positively correlated with the DS. Yet, in most cases, a significant mismatch between the model input and the simulated channels is noticed. For example, the ASD shows no dependency on the KF in the model input in Scenario 1 and Scenario 3 and has a positive correlation of 0.25 in Scenario 2. However, the values calculated from the simulated channels show a strong positive correlation in all scenarios. Similar low agreement is noticed in the ASD-SF and ASD-DS correlation values. This mismatch could be partially explained by the previously mentioned difference in the scattering model and the estimation error of the angular spreads.

It should be noted that the cross-correlations are computed over the complete dataset without separating LoS, OLoS, and NLoS conditions. This is because the LoS segments are too short in most scenarios to provide sufficient samples for a reliable estimate, and splitting the dataset was found to cause the distributions to deviate from log-normal behavior. The separation by propagation condition is therefore applied only to the DS and KF, as discussed in Section 4.1.

Another factor that contributes to the mismatch is related to the channel simulation. QuaDRiGa applies the cross-correlation matrix simultaneously across all LSPs when generating the 2D parameter maps. This can introduce additional coupling between parameters that goes beyond what was observed in the measurements.

### 5.7. Link-Level Performance Evaluation

In order to develop a reliable communication system and evaluate its performance, realistic channel models are required. The following will investigate this statement through link-level simulations.

The aim of the simulations is:

1. To show the effect of the channel model on the link-level performance.
2. To further validate the proposed V2VRU channel model.

The link-level simulator developed by u-blox, known as the Ublox-V2X simulator [46], is utilized. This simulator is an open-source MATLAB (R2021b) model designed to assess the performance of IEEE 802.11p in terms of packet error rate (PER). The evaluation is conducted across various signal-to-noise ratio (SNR) levels, employing two modulation and coding schemes (MCSs): BPSK 3/4 and 16-QAM 3/4. For the simulations, a packet size of 350 bytes is chosen following the recommendation of the European Telecommunications Standards Institute (ETSI) in [47], with further details available in [48].

PER is defined as the ratio of the number of erroneously received packets to the total number of transmitted packets. Lower PER values indicate better performance. In our simulations, 100,000 frames were transmitted for each SNR value. For instance, a PER curve for 10 SNR values is based on 1 million transmitted frames, with a new channel realization with unity gain generated for each frame.

To demonstrate the impact of the channel model on link-level performance, communication performance is analyzed using our WINNER-type GSCMs developed for V2VRU communication, along with two tapped-delay line (TDL) channels and an AWGN channel. The two TDL models were developed for urban V2V scenarios and have been adopted by ETSI for V2V communications [49]. The first scenario involves an urban LoS where two vehicles approach each other in an urban environment with maintained LoS. The second scenario is an urban NLoS, where two vehicles approach an intersection from different directions with LoS blocked by buildings. More details about these TDL models can be found in [48].

To further validate our proposed channel model, the PER was also evaluated using the measured V2VRU channel and compared with the PER achieved using the proposed model.

**Figure 11:**
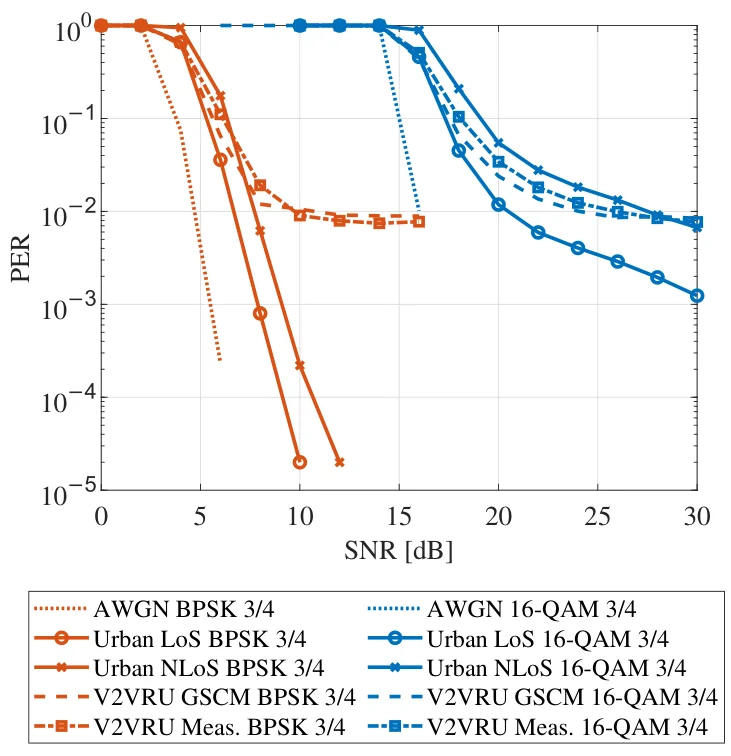
Packet error rate versus signal to noise ratio for Scenario 2 for two MCSs and different fading channel models.

In Figure 11, we plot the PER versus SNR for all channel models and with two MCSs for Scenario 2. In this scenario, the cyclist, as illustrated in Figure 3b, moves alongside the vehicle towards the intersection’s collision point. The PER is inversely proportional to the SNR. It is observed that PER performance worsens when a higher MCS is used while keeping the SNR constant. Consequently, a higher SNR is needed to achieve the same performance when transitioning from a lower to a higher MCS order. Compared to BPSK 3/4, PER decreases more slowly with increasing SNR for 16-QAM 3/4.

As expected, the AWGN channel (dotted line) outperforms the fading channels. When comparing performance using the Urban LoS and Urban NLoS V2V TDL channels (circled and crossed lines), it is clear that the Urban LoS channel is less challenging, providing better performance due to the strong LoS and lower delay spread. The LoS channel’s performance is similar to that of the GSCM at low SNR levels, specifically below 5 dB and 17 dB with BPSK 3/4 and 16-QAM 3/4, respectively. However, the GSCMs reveal that PER does not decrease with increasing SNR, resulting in an error floor. Unlike AWGN and TDL channels, an error-free link is unattainable for the V2VRU GSCMs regardless of SNR. This error floor may be mitigated by enhancing receiver design and implementing more efficient channel estimation techniques. Investigating mitigation strategies for this error floor is outside the scope of this channel modeling study; however, the proposed GSCM can be used for this purpose in future work.

When comparing the PER of the measured channel with that of other channel models, our GSCM provides the closest match. This emphasizes the importance of using a dedicated and realistic GSCM to accurately evaluate communication system performance.

### 5.8. Limitations of the Proposed Model

First, the campaign was conducted at a single urban location in Germering near Munich. The site was selected because it combines the elements that characterize the critical accident scenarios reported in [2,3], an intersection with building blockage, parked vehicles along both sides of the street, and the resulting LoS/OLoS/NLoS transitions. The measured scenarios therefore cover the propagation mechanisms relevant for V2VRU links. However, a single location cannot capture the variability of all urban environments. Differences in street width, building geometry, parked-vehicle density, and road layout can lead to different channel statistics. Consequently, the reported parameters are representative of the specific urban scenarios examined in this study, and should not be interpreted as universally applicable to all urban V2VRU environments.

Second, the measurements were performed in a SISO configuration with vertically polarized omnidirectional antennas. The AoD and AoA were not measured directly but derived from localized last-bounce scatterers under a single-bounce assumption. This indirect estimation introduces additional uncertainty in the angular spreads and their cross-correlations, which is evident from the ASA mismatch in Scenario 1 (Section 5.5). Moreover, the present configuration cannot resolve double-bounce scattering, elevation spreads, or any polarization-dependent effects, all of which are omitted from the current model. While the path-loss exponent, SF, DS, and KF are unaffected by these angular limitations and can be applied directly to single-antenna link- and system-level analyses, extending the model to multiple-input-multiple-output (MIMO) scenarios will require dedicated multi-antenna measurements that explicitly capture AoD/AoA, elevation, and polarization characteristics.

Third, the number of measurement runs differs between scenarios. Scenarios 1 and 3 contain 10 and 9 runs, respectively, and Scenario 2 only two. The effect of the limited number of runs on the Scenario 2 parameters was therefore examined. The LSPs of Scenario 2 were re-estimated separately for each run, using the same procedure as for the model input. Table 9 compares them with the values of Table 6. The per-run means of the SF differ from the model input by at most 0.12 dB, and those of the DS and the angular spreads by at most 0.08 in log-domain units. The corresponding differences in the standard deviations stay below 0.07. The right part of Table 9 reports the spread of the per-run means, i.e., their standard deviation across the runs of a scenario, for all three scenarios. For Scenario 2, the spread is at most 0.17 dB for the SF and 0.10 in log-domain units for the DS and the angular spreads, which is of the same order as in Scenarios 1 and 3 and smaller in most cases. The two runs therefore yield consistent estimates for these parameters. The LoS KF is the exception, with a spread of 2.49 dB, corresponding to a difference of 3.5 dB between the two runs. This is attributed to the short LoS segment, which covers only the last few meters before the collision point and is sensitive to the position at which the direct path clears the last parked vehicle. The LoS KF of Scenario 2 should therefore be regarded as preliminary.

Fourth, the measurements were performed at 5.2 GHz rather than in the 5.9 GHz ITS band used by ITS-G5 and by LTE-V2X and NR-V2X sidelink. At 5.9 GHz, the free-space path loss increases only marginally, by approximately 1.1 dB, while the path loss exponent remains unchanged. For the delay spread, the frequency dependence specified for urban scenarios in Table 7.5-6 in TR 38.901 [50] results in a small difference between 5.2 GHz and 5.9 GHz, which is considered smaller than the variability observed in the measurements. The obstruction geometry is the same at both frequencies. The Fresnel–Kirchhoff diffraction parameter increases with $\sqrt{f}$, so the small frequency shift leads to only a slight change in the diffraction conditions. Validation at 5.9 GHz and in the other bands used by LTE-V2X and NR-V2X remains future work.

Fifth, the DMCs are included only through their power contribution to the path loss models. The DS, KF, and angular spreads are estimated from the SMCs. Since the diffuse power is distributed over larger delays, the DS is underestimated and the KF overestimated relative to values that include the diffuse contribution. At the link level, the delay spreads are well below the cyclic prefix duration of 1.6 μs, so omitting the DMC contribution is not expected to cause significant inter-symbol interference. The main effect of the DMC on the PER is therefore through the fading statistics. The higher K-factor of the SMC-based model corresponds to slightly less severe fading than in the measured channel. The DMC characteristics of this measurement campaign were analyzed separately in [32]. A stochastic model of the DMCs and their incorporation into the LSP estimation are left for future work.

Finally, the VRU trajectories were controlled and do not cover the full range of natural pedestrian and cyclist behavior. Variations in speed, direction, and lateral movement may produce additional channel variability.

## 6. Conclusions and Future Work

V2VRU communication overcomes the limitations of sensor-based collision avoidance systems and provides 360° of awareness by exchanging information between vehicles and VRUs. In order to develop a reliable V2VRU communication system, realistic channel models covering relevant accident scenarios are of immense importance. For this purpose, a SISO channel measurement campaign was conducted using the RUSK-DLR channel sounder at a carrier frequency of $f_{c}=$ 5.2 GHz and a bandwidth of B= 120 MHz. The measurement campaign was conducted in an urban environment. The three most critical accident scenarios involving pedestrians and cyclists were considered.

A measurement-based parameterization of the WINNER-type GSCM large-scale parameters was proposed. The LSPs were estimated in the power and delay domain, i.e., SF, rms DS, and narrowband KF, and in the angular domain, i.e., ASD and ASA. The log-normal distribution was found to provide a good fit to the distributions of the LSPs. Furthermore, the spatial correlations of the LSPs were analyzed and their correlation distances were calculated. In order to ensure the channel spatial consistency, the cross-correlation coefficients among the LSPs were also calculated.

The proposed model parameters are used as an input to the QuaDRiGa channel simulator. The proposed channel model is then validated by comparing the simulated channels with the measured channels. It can be concluded that the GSCM with the proposed model parameters is able to produce similar statistics as the measured channels. The proposed model reproduces the main large-scale statistical characteristics of the measured V2VRU channel in the considered scenarios, with the strongest agreement observed in the power- and delay-related parameters. It can therefore serve as a realistic channel model for the simulation-based evaluation of V2VRU communication systems and related safety applications in critical accident scenarios.

Furthermore, we have investigated the impact of the channel model on the performance of the communication system through link-level simulations. The results reveal the challenges that V2X communication systems have to face in urban environments: namely an error floor despite a high SNR. Using the proposed GSCM in link-level evaluations provides a more realistic performance compared to the optimistic performance obtained by the TDL channels.

Several directions remain open for future work. First, the model is limited to SISO and extending it to MIMO requires multi-antenna measurements to resolve double-bounce scattering. Second, the VRU mobility model assumes constant speed along a fixed trajectory, which may not always reflect real pedestrian behavior. Finally, validating the model at other frequency bands, particularly those relevant to 5G NR-V2X, is an important next step.

## Figures and Tables

**Table 1:** Comparison of existing V2VRU channel measurement studies with this work.

Ref.	$f_{c}$/BW	Meas. Type	Conditions	Parameters Reported
[24]	3.8 GHz/200 MHz	Wideband	LoS	PL, LSF, SSF
[25]	3.8 GHz/200 MHz	Wideband	LoS/NLoS	PL, LSF, SSF
[26]	3.8 GHz/200 MHz	Wideband	LoS, partial LoS	ACF
[27]	5.8 GHz/n/a	Narrowband	Business district	RSSI, KF
[28]	5.8 GHz/n/a	Narrowband	LoS	PL, LSF, SSF
This work	5.2 GHz/120 MHz	Wideband	LoS/OLoS/NLoS (buildings, parked cars)	PL, SF, DS, KF, ASD, ASA, $d_{c}$, cross-corr.

**Table 2:** Channel sounder parameters in the urban measurements.

Parameter	Value
RF center frequency $f_{c}$	5.2 GHz
Bandwidth *B*	120 MHz
Transmit power $P_{T}$	37 dBm
Signal period $T_{p}$	3.2 μ s
Time grid $T_{g}$	1.024 ms
Tx antenna	Omni-directional (V-polarized), 8 dBi
Rx antenna	Omni-directional (V-polarized), 8 dBi
Vehicle speed	5–11 m s^−1^
Cyclist speed	1.5 m s^−1^
Pedestrian speed	1 m s^−1^

**Table 3:** Estimated scaling coefficients in all scenarios.

Scaling Coefficient	Scenario 1	Scenario 2	Scenario 3
$g^{\mathrm{DS}}$	1.97	2.71	3.48
$g^{\mathrm{ASD}}$	2.57	2.35	1.82
$g^{\mathrm{ASA}}$	1.98	2.38	2.18

**Table 4:** Log-distance path loss model parameters for the three scenarios.

	LoS			OLoS/NLoS				
	Slope 1			Slope 1			Slope 2	
	*P*_L_ (*d*_0_) [dB]	*n*	*σ* [dB]	*P*_L_ (*d*_0_)	*n*	*σ*	*n*	*σ*
Scenario 1	55.70	1.40	1.90	−15.16	6.90	–	2.90	3.46
Scenario 2	49.46	1.70	1.53	44.37	2.40	1.88		
Scenario 3	49	1.80	3.40	31.97	3.20	5.79		

**Table 5:** Comparison of large scale parameters (LSPs) for Scenario 1.

Parameters		Model Input		Simulated Channels	
		LoS	NLoS	LoS	NLoS
SF [dB]	μ	0	0	0	0
	σ	3.20	3.20	3.35	3.35
	$d_{c}$ [m]	8.20	8.20	10.33	10.33
	RMSE	0.007	0.007	0.015	0.015
K-factor [dB]	μ	15.02	N/A	14.44	N/A
	σ	4.81	N/A	4.98	N/A
	$d_{c}$ [m]	8.01	N/A	8.83	N/A
	RMSE	0.024	0.024	0.031	0.031
DS [$\log_{10}\left(s\right)$]	μ	−8.13	−7.44	−8.08	−7.46
	σ	0.34	0.16	0.31	0.19
	$d_{c}$ [m]	8.97	49.69	9.08	50.16
	RMSE	0.025	0.017	0.036	0.020
ASD [$\log_{10}\left({}^{\circ}\right)$]	μ	0.74	0.74	0.89	0.89
	σ	0.50	0.50	0.29	0.29
	$d_{c}$ [m]	37.11	37.11	52.04	52.04
	RMSE	0.017	0.017	0.097	0.097
ASA [$\log_{10}\left({}^{\circ}\right)$]	μ	1.02	1.02	1.40	1.40
	σ	0.40	0.40	0.25	0.25
	$d_{c}$ [m]	48.95	48.95	55.71	55.71
	RMSE	0.011	0.011	0.159	0.159

**Table 6:** Comparison of LSPs for Scenario 2.

Parameters		Model Input		Simulated Channels	
		LoS	OLoS	LoS	OLoS
SF [dB]	μ	0	0	0	0
	σ	1.80	1.80	2.82	2.82
	$d_{c}$ [m]	2.27	2.27	5.54	5.54
	RMSE	0.006	0.006	0.021	0.021
K-factor [dB]	μ	16.22	5.34	15.90	5.93
	σ	2.88	4.38	2.87	4.80
	$d_{c}$ [m]	4.42	30.58	4.57	46.05
	RMSE	0.014	0.019	0.015	0.038
DS [$\log_{10}\left(s\right)$]	μ	−8.19	−7.60	−8.10	−7.62
	σ	0.19	0.21	0.18	0.21
	$d_{c}$ [m]	4.42	70	4.57	59.45
	RMSE	0.032	0.020	0.090	0.034
ASD [$\log_{10}\left({}^{\circ}\right)$]	μ	0.86	0.86	0.86	0.86
	σ	0.39	0.39	0.32	0.32
	$d_{c}$ [m]	63.23	63.23	66.03	66.03
	RMSE	0.014	0.014	0.021	0.021
ASA [$\log_{10}\left({}^{\circ}\right)$]	μ	1.03	1.03	1.14	1.14
	σ	0.39	0.39	0.26	0.26
	$d_{c}$ [m]	66.17	66.17	71.48	71.48
	RMSE	0.010	0.010	0.057	0.057

**Table 7:** Comparison of LSPs for Scenario 3.

Parameters		Model Input		Simulated Channels	
		LoS	OLoS	LoS	OLoS
SF [dB]	μ	0	0	0	0
	σ	4.69	4.69	6.31	6.31
	$d_{c}$ [m]	7.60	7.60	16.43	16.43
	RMSE	0.012	0.012	0.031	0.031
K-factor [dB]	μ	17.32	6.44	16.76	7.18
	σ	4.09	5.31	4.42	5.36
	$d_{c}$ [m]	4.23	26.97	3.07	40.89
	RMSE	0.019	0.012	0.026	0.024
DS [$\log_{10}\left(s\right)$]	μ	−8.09	−7.62	−8.07	−7.62
	σ	0.23	0.22	0.23	0.20
	$d_{c}$ [m]	4.64	57.88	3.19	54.60
	RMSE	0.004	0.017	0.010	0.016
ASD [$\log_{10}\left({}^{\circ}\right)$]	μ	1.04	1.04	1.02	1.02
	σ	0.30	0.30	0.30	0.30
	$d_{c}$ [m]	55.61	55.61	56.24	56.24
	RMSE	0.013	0.013	0.012	0.012
ASA [$\log_{10}\left({}^{\circ}\right)$]	μ	1.11	1.11	1.16	1.16
	σ	0.33	0.33	0.28	0.28
	$d_{c}$ [m]	54.48	54.48	58.12	58.12
	RMSE	0.010	0.010	0.027	0.027

**Table 8:** Comparison of cross-correlation coefficients for the three scenarios.

Cross-Correlation		Model Input					Simulated Channels				
		SF	KF	DS	ASD	ASA	SF	KF	DS	ASD	ASA
Scenario 1	SF	1	0.50	−0.42	0.28	−0.07	1	0.61	−0.34	−0.07	−0.01
	KF		1	−0.86	0	−0.01		1	−0.78	−0.44	−0.35
	DS			1	−0.33	−0.04			1	0.09	0.48
	ASD				1	0.58				1	0.31
	ASA					1					1
Scenario 2	SF	1	0.35	−0.30	−0.21	−0.36	1	0.35	−0.30	−0.01	−0.11
	KF		1	−0.87	0.25	−0.01		1	−0.91	−0.50	−0.63
	DS			1	−0.13	0.07			1	0.47	0.58
	ASD				1	0.63				1	0.28
	ASA					1					1
Scenario 3	SF	1	0.63	−0.51	−0.07	−0.26	1	0.49	−0.49	−0.21	−0.26
	KF		1	−0.85	0	−0.1		1	−0.82	−0.48	−0.55
	DS			1	0.02	−0.07			1	0.44	0.45
	ASD				1	0.5				1	0.16
	ASA					1					1

**Table 9:** Per-run LSP estimates for Scenario 2 compared with the model input, and run-to-run spread of the per-run means in all three scenarios S1, S2 and S3.

Par.	Cond.	Run 1		Run 2		Model Input		Spread of μ		
		*μ*	*σ*	*μ*	*σ*	*μ*	*σ*	S1	S2	S3
SF [dB]	all	0.11	1.85	−0.12	1.74	0.00	1.80	1.12	0.17	2.41
KF [dB]	LoS	18.35	2.15	14.83	2.40	16.22	2.88	2.31	2.49	1.56
	OLoS	5.47	5.06	5.18	3.47	5.34	4.38	0.95	0.21	2.53
DS [log_10_(*s*)]	LoS	−8.27	0.13	−8.14	0.21	−8.19	0.19	0.10	0.10	0.13
	OLoS	−7.65	0.23	−7.54	0.18	−7.60	0.21	0.05	0.07	0.10
ASD [log_10_(°)]	all	0.82	0.38	0.90	0.39	0.86	0.39	0.07	0.06	0.08
ASA [log_10_(°)]	all	0.97	0.40	1.11	0.37	1.03	0.39	0.09	0.10	0.09

## Data Availability

The data presented in this study are available on request from the corresponding author due to ongoing developments and data-sharing restrictions.

## Abbreviations

The following abbreviations are used in this manuscript:

2D	2-Dimensional
3GPP	3rd Generation Partnership Project
ACF	Autocorrelation Function
AoA	Angle of Arrival
AoD	Angle of Departure
AS	Angular Spread
ASA	Azimuth Spread of Arrival
ASD	Azimuth Spread of Departure
AWGN	Additive White Gaussian Noise
BPSK	Binary Phase Shift Keying
CDF	Cumulative Distribution Function
CIR	Channel Impulse Response
COST	European Cooperation in Science and Technology
C-V2X	Cellular Vehicle-to-Everything
DMC	Diffuse Multipath Component
DS	Delay Spread
DSRC	Dedicated Short-Range Communication
ETSI	European Telecommunications Standards Institute
GDP	Gross Domestic Product
GDV	General Association of the German Insurance Industry
GNSS	Global Navigation Satellite System
GSCM	Geometry-Based Stochastic Channel Model
IEEE	Institute of Electrical and Electronics Engineers
ITS	Intelligent Transport Systems
KEST	Kalman Enhanced Super resolution Tracking
KF	K-Factor
LoS	Line of Sight
LSP	Large-Scale Parameter
LTE	Long Term Evolution
LSF	Large-Scale Fading
MCD	Multipath Component Distance
MCS	Modulation and Coding Scheme
MIMO	Multiple Input Multiple Output
MoM	Method of Moments
MPC	Multipath Component
NHTSA	National Highway Traffic Safety Administration
NLoS	Non-Line of Sight
NR	New Radio
OLoS	Obstructed Line of Sight
PER	Packet Error Rate
PL	Path Loss
QAM	Quadrature Amplitude Modulation
RMSE	Root Mean Square Error
RSSI	Received Signal Strength Indication
SAGE	Space-Alternating Generalized Expectation-Maximization
SCM-E	Spatial Channel Model-Extended
SF	Shadow Fading
SSF	Small-Scale Fading
SISO	Single-Input Single-Output
SMC	Specular Multipath Component
SNR	Signal-to-Noise Ratio
TDL	Tapped-Delay Line
V2I	Vehicle-to-Infrastructure
V2P	Vehicle-to-Pedestrian
V2V	Vehicle-to-Vehicle
V2VRU	Vehicle-to-Vulnerable Road User
V2X	Vehicle-to-Everything
VRU	Vulnerable Road User
WINNER	Wireless World Initiative for New Radio
WSS	Wide-Sense Stationary
